# Preface

**Published:** 1781-07

**Authors:** 


### PREFACE.

AFTER completing two volumes of their Journal, the Society find it neceſſary to apologize to their readers for making ſome alteration in the publication of a work that has been honored in a very eminent degree with the patronage of the public.

When the Society firſt engaged in this plan their object was to employ their leiſure moments in a manner that might be uſeful to themſelves and the community. They were likewiſe deſirous to co-operate with other ſimilar inſtitutions in promoting that laudable ſpirit of obſervation and inquiry which has of late years ſo happily prevailed in this country.—In theſe views the Society have not been diſappointed.—Their own induſtry has been ſtimulated to peruſe with attention a variety of medical works with which it is probable they would otherwiſe never have been acquainted; and they flatter themſelves that thoſe among their readers who want opportunity to procure, or leiſure to read many new books, eſpecially foreign ones, have received uſeful information from their labours. The number of reſpectable and ingenious practitioners, who have honoured the Society with their correſpondence, alſo affords them the moſt pleaſing pleafing fatisfaction, as they fee their Journal every day deriving additional importance from the interefting facts and remarks that are inferted under the head of *Effays and Obfervations*. Thefe would be fufficient inducements to them to proceed as they began; but a year's experience has convinced them that their periods of publication are too frequent. The gentleman who contributes the moft largely to the work, and who acts as Editor, finds the trouble of a monthly publication incompatible with his other avocations, fo that what was originally propofed as a means of filling up a few vacant hours, has been found to require more time than he can conveniently fet apart for it.

In future the Society mean to adopt a mode of publication by which this inconvenience will be avoided. The fame general plan will be purfued as before in the diftribution of their materials, but inftead of once a month, a number of their Journal will appear only once a quarter. Each number will confift of feven fheets, fo as to make one volume at the end of the year. This arrangement, it is hoped, will meet with the approbation of their readers.

*London*,
Dec. 29, 1781.

